# Benzydamine rescues ethanol-induced teratogenesis in zebrafish FASD model

**DOI:** 10.1038/s41598-025-93539-8

**Published:** 2025-03-17

**Authors:** Tiasha Dasgupta, Venkatraman Manickam, Ramasamy Tamizhselvi

**Affiliations:** Department of Biosciences, School of Biosciences and Technology, Vellore Institute of Technology, Vellore, Tamil Nadu India

**Keywords:** Ethanol, FASD, Glutathione, Oxidative stress, Malformations, Cellular damage, Developmental biology, Molecular biology

## Abstract

Fetal alcohol spectrum disorders (FASD) are a group of physical, behavioral, and cognitive impairments caused by ethanol exposure during pregnancy. Zebrafish have emerged as a useful model for researching FASD and its variants in recent years. Oxidative stress has been identified as the primary damaging pathway, notwithstanding the possibility of other mechanisms at play. In this regard it’s important to put an effort towards antioxidants which can exhibits and bare a potential to counteract the oxidative stress induced by ethanol during embryos development. Previosuly benzydamine has shown to protect macrophages against ethanol-induced condition by stabilizing redox homeostatis. This study aims to repurpose the Non-Steroidal Anti-Inflammatory Drug (NSAID) benzydamine to mitigate ethanol-induced teratogenesis during the early embryonic stage in Zebrafish. Zebrafish embryos were treated with 1% ethanol at 2 h post fertilization (hpf) and co-exposed with benzydamine (5–20 µM) after 2 h of ethanol treatment for 24 h. Reactive oxygen Species (ROS) and biochemical analysis was carried out at 48hpf. 1% ethanol significantly increased the production of ROS along with increased in lipid peroxidation followed by a decrease in glutathione (GSH) level when compoared to the control group (*P* < 0.001). These conditions were positively encountered by benzydamine (10, 15 µM) and returned to basal level. Involvement of two ethanol metabolizing enzymes cyp2y3 (Cytochrome P450, family 2, subfamily Y, polypeptide 3) and cyp3a65 Cytochrome P450, family 3, subfamily A, polypeptide 65 ) were also studied at 48hpf. 1% ethanol exposure aggregately elevated the expression of these two enzymes which showed a significant decrease in the benzydamine treated groups. Furthermore, the malformations and cellular damage due to 1% ethanol was studied at 96hpf, where 1% ethanol made severe malformation along with muscle fiber alteration, apoptosis in the brain and eye as manifested. These conditions were successfully reverted by benzydamine. In conclusion, ethanol causes oxidative stress, cellular damage along with severe malformation at early embryonic stage, which were partially prevented by the exposure of benzydamine.

**Supplementary Information:**

The online version contains supplementary material available at 10.1038/s41598-025-93539-8.

## Introduction

Ethanol like teratogen can result in multisystemic illness throughout any point in life. In the US, the prevalence of lifetime alcohol use disorders (AUD) is about 12%^1^. Ethanol exposure during early embryonic development coined as Prenatal alcohol exposure (PAE) can cause Fetal alcohol spectrum disorder (FASD)^2,3^. Growth retardation both pre- and post-natal, facial deformities (small eye openings), sensory (visual and auditory) deficiencies, poor fine motor abilities, and learning deficits, including mental retardation, are the physical and mental concerns linked to FASD^4^. Although it was traditionally believed that alcohol addiction was the cause of FASD however smaller doses of alcohol may potentially have an effect on the fetus. Lower dosages and/or shorter exposure periods to alcohol during pregnancy may have relatively mild effects known as alcohol-related birth defects (ARBD) or alcohol-related neurodevelopmental disorder (ARND)^5^. Various mechanism has been reported behind ethanol teratogenicity, among which reduction in the antioxidant capacity endogenously either by lowering glutathione peroxidase or by producing free radicals and reactive oxygen species^6^considered to induce uncontrolled apoptosis in the brain and other organs^7^.

There are several drawbacks to research that use humans to examine the teratogenic consequences of ethanol, such as the inability to regulate dosage, exposure time, or response metrics^8^with no distinct treatment or precautionary strategies to diminish teratogenic effects induced by ethanol^9^. However, to understand FASD in human various research has been done on zebrafish embryos as zebrafish and humans have significant levels of evolutionary conservation^10^. Zebrafish also present a number of unique benefits for genetic and embryological research due to external fertilization, fast development, ease of genetic manipulation, high fecundity, and optical clarity of the embryo made them as an alternative model to study FASD. An additional advantage of this model is that the eggs are transparent, and phenotypic changes at particular embryonic stages can be easily identified without interfering with the process of development. The transparency of zebrafish eggs and the ability of alcohol to pass through the chorion make them an ideal model for studying embryonic development^11^. Furthermore, because zebrafish develop quickly (3 months to adult stage), it is possible to compare a few generations in a time interval that is significantly shorter than that of other animal models^12^. This allows researchers to examine the effects of alcohol on subsequent generations as well as different combinations of concentration and exposure time period of ethanol. Moreover, Exposure of zebrafish embryos to ethanol results in growth deficiencies throughout pre- and post-hatching, as well as phenotypic abnormalities resembling those of children with FASD. These findings indicate that the same molecular pathway is harmed in both humans and zebrafish when exposed to ethanol^13–15^.

To combat ethanol-induced teratogenicity several techniques have been proposed yet there are no precise therapies or preventive measures are promising until today. One of the most often recommended medication classes for people with inflammation is nonsteroidal anti-inflammatory drugs (NSAIDs). Previous studies has shown that ethanol-induced teratogenicity is mostly due to increased in the oxidative strss followed by activation of several pro-inflammatory mediators. In recent times the craze behind repurposing NSAIDs has increased tremendously. The goal of drug repurposing (also known as drug repositioning) is to repurpose a medication that has undergone rigorous safety and effectiveness assessment for a different or supplementary use^16^.

Benzydamine, an indazole derivative of the NASID class exhibits anti-inflammatory activity, however, when applied topically, benzydamine’s analgesic/anaesthetic properties can be fully utilized to provide it a competitive edge over NSAIDs^17^. Like other NSAIDs, benzydamine’s mechanism of action isn’t directly involved decrease in prostaglandin E2 synthesis (PGE2) followed by cyclooxygenase-2 (COX-2) inhibition^18^. Previous studies showed benzydamine inhibits the release of pro-inflammatory cytokine tumour necrosis factor-α (TNF- α), thus leading to decrease in COX-2 activity and eventually decreased synthesis of PGE2^19,20^. Previously in our study we have shown with ethanol exposed macrophages, benzydamine decreased expression of pro-inflammatory cytokines, and inhibited NF-κB (Nuclear Factor-Kappa B) translocation to the nucleas. Benzydamine at a very lower dosage decreased ROS generation along with stabilized MMP in ethanol exposed macrophages^21^. This was the first study where we have reported the protective role of benzydamine against ethanol-induced oxidative stress and balancing cellular homeostatis. This previous study has drove us to explore more on benzydamine’s role against ethanol induced condition. Thus this study aims to explore the preventive measurement of benzydamine against ethanol-induced toxicity in zebrafish embryos, an alternative effective animal model.

## Materials and methods

### Materials

Benzydamine Hydrochloride was purchased from Sigma, India. Dichlorofluorescin diacetate, Reduced Glutathione, Thiobarbituric acid, and Molecular Biology Grade Ethanol were obtained from Hi-Media, India.

### Zebrafish maintenance and embryos collection

The collection of adult zebrafish was done from a local vendor (Chennai, India). Wild Type (WT) zebrafishes were maintained in 12/12 h light and dark conditions at 26 ± 2 °C.They were fed two times a day with commercially available food supplements (Taiyo grow) along with live brine shrimps. After 1 week of acclimatization, using the natural swapping method (2 male: 1 female) early in the morning, the embryos were collected within 30 min after switching on the light^3^. All the experiments were conducted with similar batch of male and female fishes. After collecting the embryos, they were washed thoroughly in E3 media (5.03 mM NaCl, 0.17 mM KCL, 0.33 mM CaCl_2_.2H_2_O, 0.33 mM MgSO_4_.7H_2_O). After that, the embryos were segregated into different Petri dishes.

#### Ethical approval

All the experiments were conducted according to the *Institutional Animal Ethical Committee (VIT/IAEC/22/Dec 22/09)*, Vellore Institute of Techonology, Vellore, India. All experments were carried out based on guidance and regulations.

### Ethanol exposure and benzydamine treatment

**Fig. 1:**
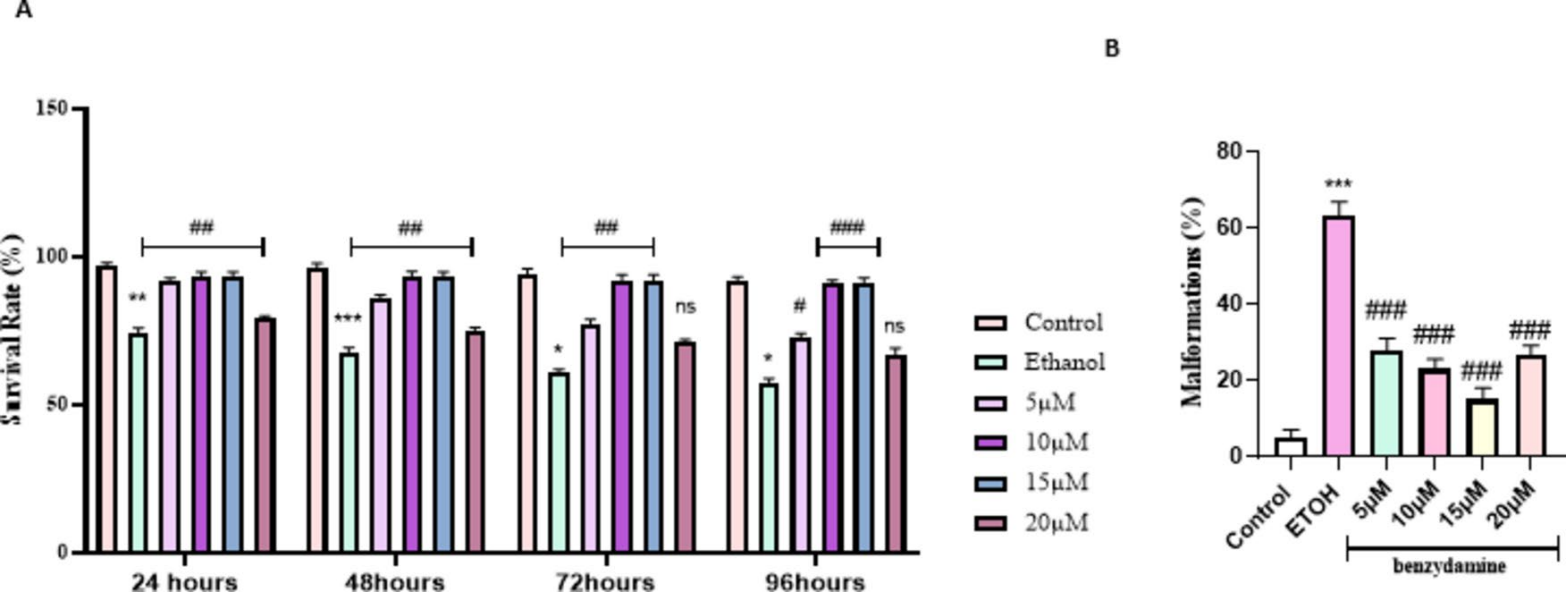
Percent survival rate and malformations of zebrafish larvae (96 hpf) after exposure to 1% ETOH and in combination with benzydamine. (A) Percent survival curves for the control, ETOH and ETOH treated with different concentration of benzydamine group. symbols in each line represents the time point with death registered for each group. (B) total malformations. Data represented as mean ± SD of three replicates. (*N* = 30 per replicates) *** *P* < 0.001 vs. control group; **P < 0.01 vs. control-treated group, *P < 0.05 vs. control-treated group ; ###*P* < 0.001 vs. ethanol-treated group, ##*P* < 0.01 vs. ethanol-treated group, #*P* < 0.05 vs. ethanol-treated group.

After approximately 2hpf, collected embryos were randomly divided into 6 well plates containing 30 embryos per well. The embryos were then subjected to 1% ethanol and co-exposed with benzydamine (5–20 µM) for 24 h. We have selected the dose based on benzydamine toxicity on zebrafish embryos (Supplementory Fig. 1). It is important to note that the administration of benzydamine was done after 2 h of ethanol instruction to the system to reduce possible drug substance interaction. After 24 h of co-exposure, the embryos were transferred to new 6-well plates and washed thoroughly to remove the traces of ethanol. The embryos were then maintained in fresh E3 media. Cultured media was changed every 24 h till 96 hpf. Each experiment was repeated at least three times^9^.

**Figure:**
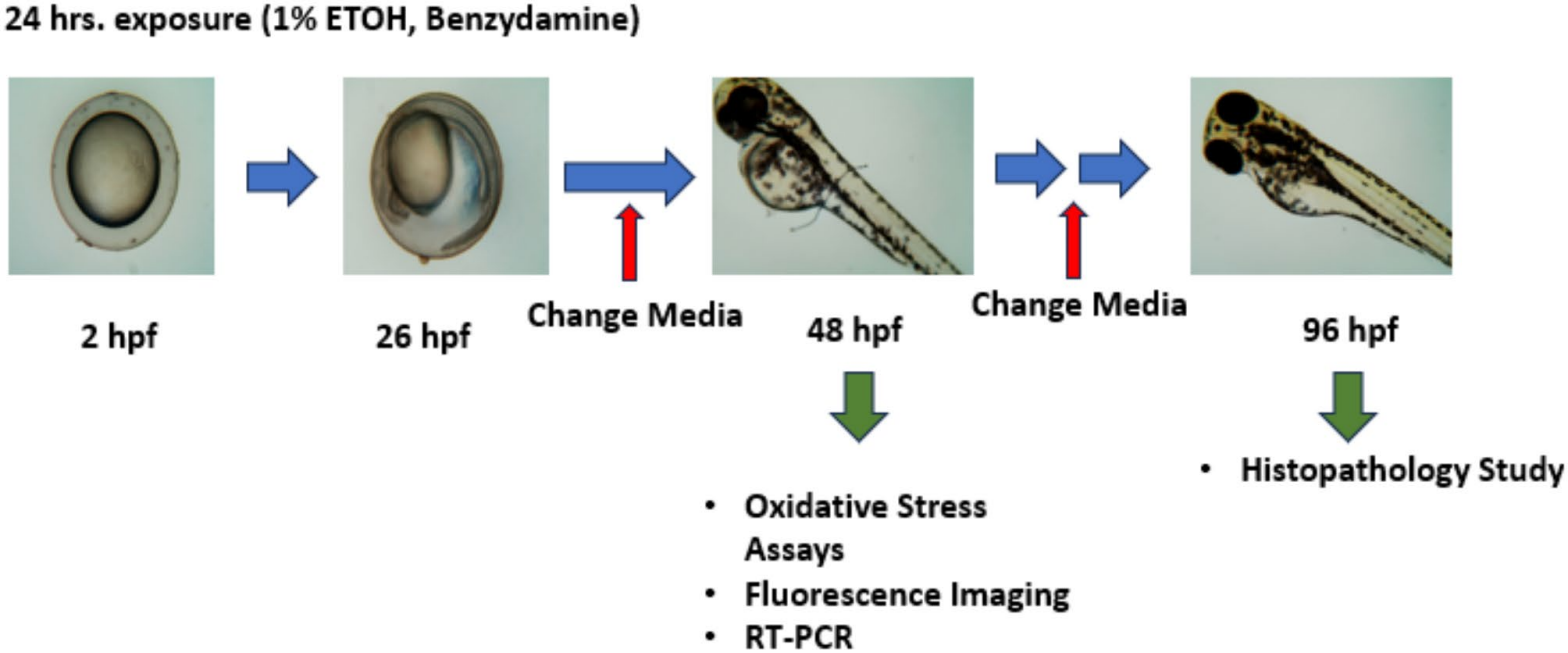
Pictorial representation of experimental design

###  Intracellular ROS generation

DCFDA dye (Dichlorofluorescin diacetate) was used to measure the production of ROS in zebrafish embryos^22^. Embryos were treated with 1% ethanol for 24 h with or without benzydamine (10 and 15 µM). After the mentioned period the embryos were kept till 48hpf. For the assay, 10 zebrafish embryos were taken and transferred to a 24-well plate. The embryos were washed thoroughly with PBS and incubated with 10 µl of DCF-DA dye (1 mg/2 ml) in 500 µl of PBS and incubated in the dark at room temperature for 30 min. Next, treated embryos were washed with 1X PBS twice and anaesthetized with ice-cold water before capturing. Images were taken in an EVOS fluorescence microscope (EVOS M5000 Imaging System). The intensity of the fluorescence was measured and calculated in ImageJ software.

### Estimation of protein

Zebrafish embryos were homogenized and protein concentration was determined using BCA kit (Takara, Japan). Absorbance was measured at 630 nm using a microplate reader (Bio-Tek, USA). Bovine Serum Albumin was used as a standard.

### LPO assay

One of the promising oxidative stress biomarkers is lipid peroxidation which produces a reactive end product malonaldehyde. TBRAS (Thiobarbituric acid reactive substances) assay was obtained to determine the concentration of malonaldehyde in ethanol-exposed zebrafish embryos. For this assay, the above-mentioned period was followed and around 20 embryos were homogenized with ice-cold potassium phosphate buffer (100 µl). 100 µl of 5% TCA (Trichloroacetic Acid) was added to the homogenate and incubated in ice for 15 min. After the incubation period, 100 µl of 0.67% TBA (Thiobarbituric Acid) was added to the mixture and centrifuged at 2000 g for 15 min at 4 °C. Later 250 µl of the supernatant was taken and boiled for 20 min and cooled it to room temperature. The absorbance was measured at 535 nm in a microplate reader (Bio-Tek, USA)^23^.

### GSH assay

For this assay the above-mentioned period was followed. Reduced glutathione level was measured in 20 embryos. Embryos were homogenate in 100 µl of ice-cold PBS (pH 7.4) and the resultant homogenate was incubated with 100 µl of 25% TCA and centrifuged for 10 min at 3000 g at 4 °C. From the mixture around 150 µl was taken and subsequently 1 ml of 60 µM DTNB and 500 µl of 50 mM potassium phosphate buffer was added. The absorbance was read at 412 in a microplate reader (Bio-Tek, USA)^24^.

### Isolation of RNA and qRT-PCR

Zebrafish embryos were treated with ethanol with or without benzydamine for 24 h. For RNA around 20 embryos were taken after 48hpf. RNA was isolated with the Trizol method. The quality of the RNA was checked in agarose gel and quantified using nanodrop (BioSpectrometer^®^, Eppendorf). Isolated RNA was converted to cDNA using PrimeScript™ RT Reagent Kit (Perfect Real Time). Obtained cDNA was amplified using Real-Time PCR master mix SYBR^®^ Premix Ex TaqTM II (Tli RNase Plus) with proper forward and reverse primers (Table 1) with beta-actin as the internal standard. The 2^−ΔΔCt^ method was used to calculate the fold change in comparison to the control. The sequence of the primers used is listed in Table 1.

**Table 1:** List of primers.

Gene	Forward Primer	Reverse Primer	Gene ID
*Beta-Actin*	5’-AAGCAGGAGTACGATGAGTC-3’	5’-TGGAGTCCTCAGATGCAT TG-3’	57,934
*cyp3a65*	5’- AAACCCTGATGAGCATGGAC-3’	5’- CAAGTCTTTGGGGATGAGGA-3’	553,969
*cyp2y3*	5’-TATTCCCATGCTGCACTCTG-3’	5’- AGGAGCGTTTACCTGCAGAA − 3’	Uniport ID: Q4VBR9

### Histopathology

For histopathological analysis, embryos were grown till 96hpf. Embryos treated with ethanol in the presence and absence of benzydamine were collected and fixed in formalin. Fixed embryos were subjected to ethanol dehydration in descending order. After being cleaned with xylene, the embryos were implanted in paraffin wax blocks. Using microtome, 5–6 μm sections were created of the paraffin blocks and placed in glass slides which were subjected to hematoxylin and eosin staining. Later on, prepared slides were visualized under an inverted microscope.

### AO staining

For apoptosis analysis, the embryos were washed with 1X PBS and incubated with 1 ml Acridine Orange (AO) (10 µg/mL) for 30 min. After the incubation time embryos were washed with 1X ice cold PBS for three times and analysed using EVOS fluroscence microscope (EVOS M5000 Imaging System).

### Statistical analysis

GraphPad Prism 8 was used to do statistical analysis. Two-way ANOVA with Bonferroni post-test was obtained to calculate the significant differences between two parameters like dosage of ethanol and time intervals. One-way ANOVA with Bonferroni post-test was done to calculate differences between the groups. Data in the study were represented as mean ± SD. *P* < 0.05 was considered as statistically significant. Each experiments were repeated at least 3 times.

## Results

### Benzydamine co-administration ameliorates ethanol-induced mortality and malformation in zebrafish embryos

The malformations and mortality due to ethanol were monitored at four different time points (24, 48,72 and 96 hpf) as shown in Fig. 1A. 1% ethanol has shown a significant mortality rate compared to control at the first 24 h of exposure among zebrafish embryos. Upon co-exposure to benzydamine, no cumulative significant mortality was recorded in the first 24 h. The different doses of benzydamine toxicity were assessed previously (Supplementary Fig. 1). After embryos were treated with ethanol during gastrulation and somitogenesis period (3 to 24 hpf) the ethanol-containing media was removed and replaced with regular embryo medium for the next three time points and observed for the malformation and mortality. Ethanol induction reduced the number of viable embryos after 48 h with an increase in the percentage of malformations (Fig. 1B), co-administration of benzydamine’s rescue towards the mortality differed in different doses at 48 h. Three doses of benzydamine ( 5, 10 and 15 µM) protected the embryos from the toxicity of ethanol significantly whereas the higher dose of benzydamine (20 µM) was shown to reduce the viable embryos at 48 hpf significantly. The mortality of zebrafish embryos was recorded at 96 hpf. The mortality rate in 5 µM benzydamine treatment after 48 hpf has increased significantly with a greater number of malformations, at the same time 10 and 15 µM continued to be protective till 96 hpf with fewer malformations in comparison to the ethanol-treated group. On the other hand, 20 µM failed to rescue ethanol-treated embryos at the end of the experiment.

### Ethanol-induced FASD-like developmental defects were rescued by benzydamine

**Fig. 2:**
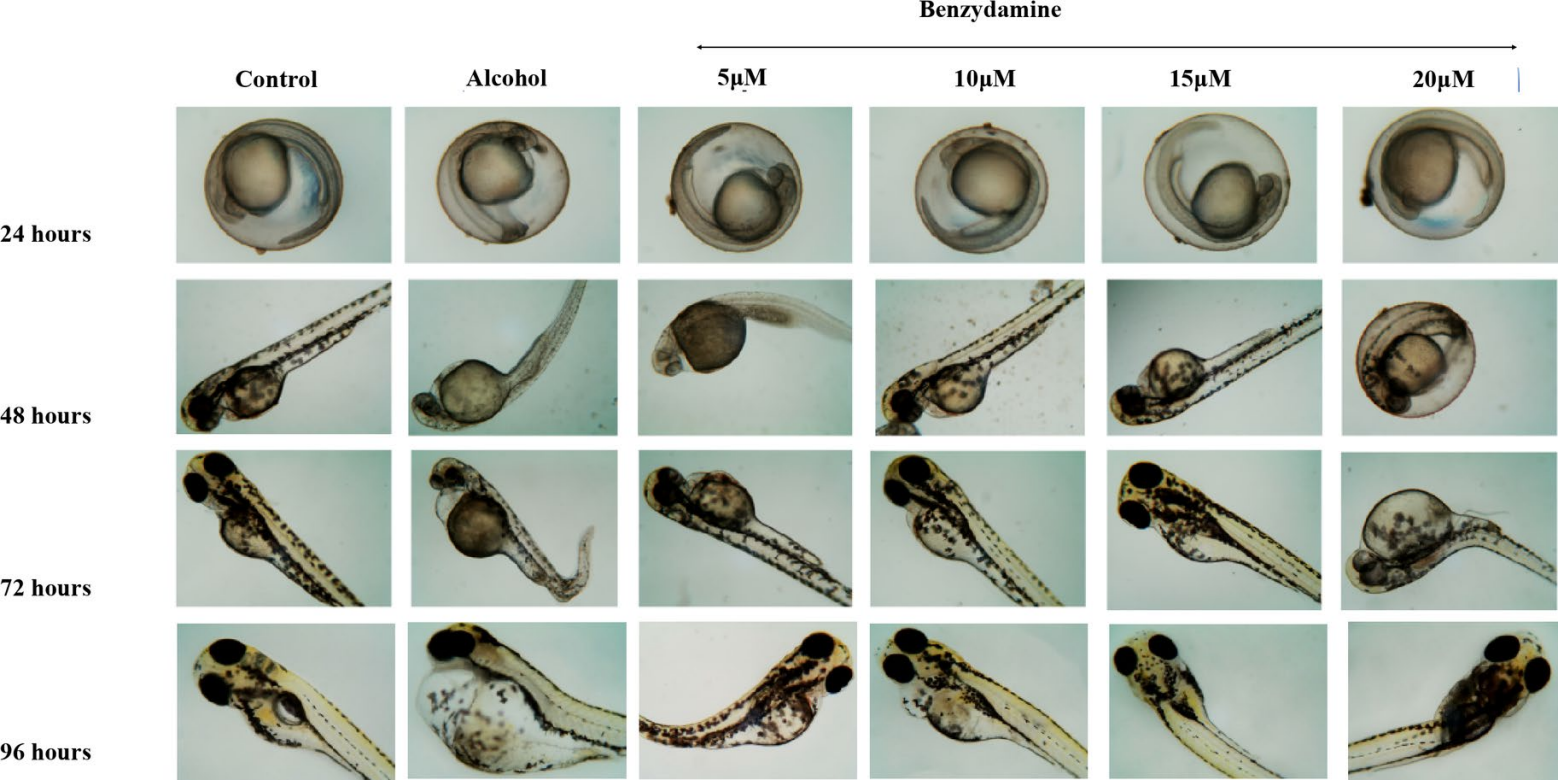
Morphological malformation of zebrafish larvae after exposure to 1% Ethanol and in combination with benzydamine different concentration at 24, 48, 72 and 96 hpf time intervals. Scale Bar – 20 μm. *N* = 30 per group.

Zebrafish embryos were monitored to examine the developmental defects induced by ethanol at four different time points. As shown in Fig. 2 ethanol with or without benzydamine didn’t alter any visible development in the first 24 hpf. After the mentioned window period of ethanol exposure, at 48 hpf, morphologically, the ethanol-treated group showed a fusion of eyes, along with an increase in the yolk area and pericardial edema. Co-administration of benzydamine at 5 to 15 µM significantly rescued the above-mentioned parameters. However, the higher concentration of benzydamine (20 µM) didn’t alter these parameters administrated by ethanol which co-relates with the increase in mortality rate, thus suggesting the co-administration with ethanol at higher doses can be fatal. Embryos were observed for another 48 h to confirm FASD-like developmental defects.

**Fig. 3:**
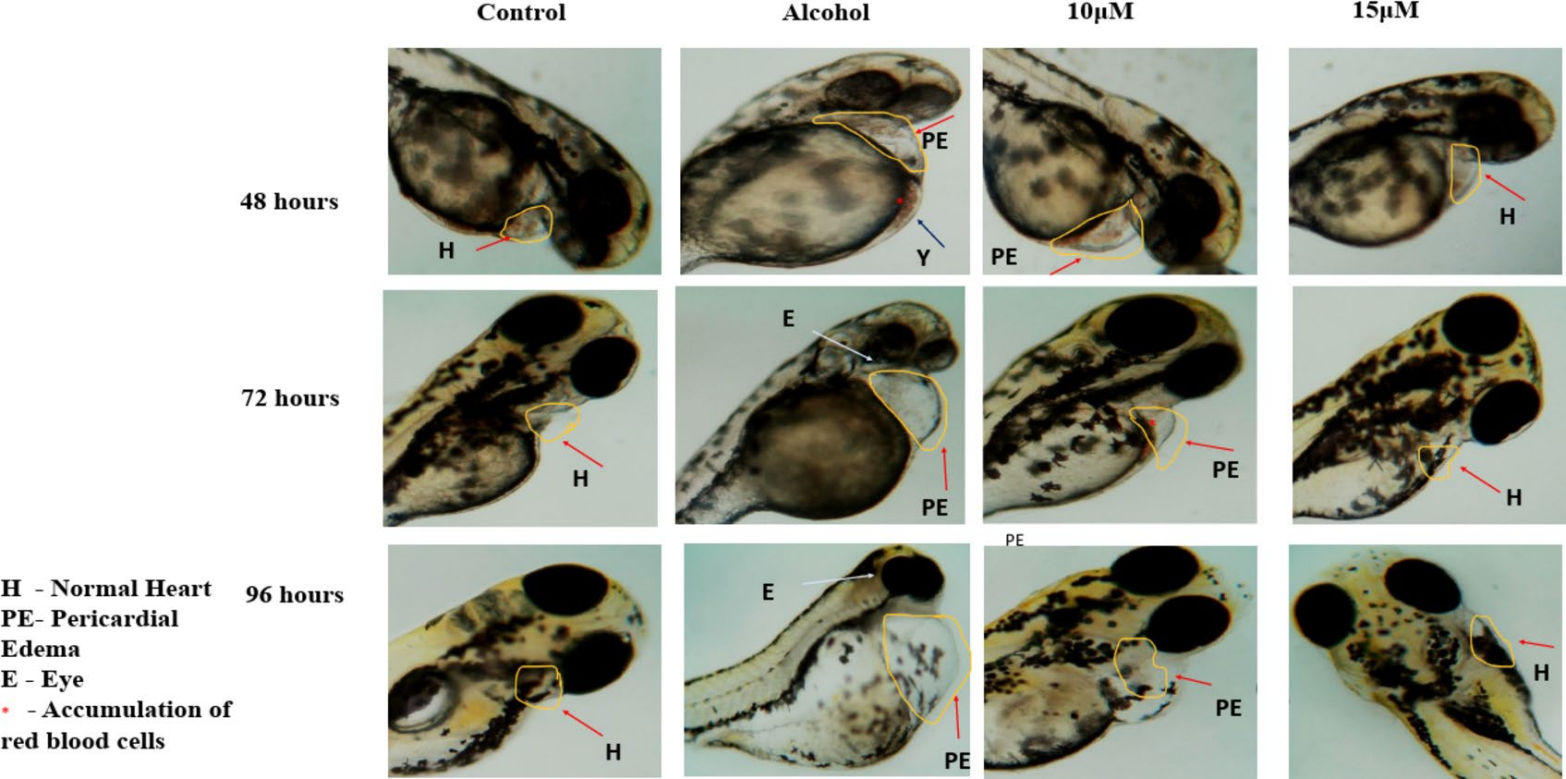
Malformations were observed after exposure to 1% ETOH named as abnormal yolk (Y), eye (E) and pericardiac oedema (PE).*N* = 30 per group.

FASD-like features in zebrafish embryos were evaluated with three major parameters including large Yolk Area (Y), Pericardial Edema (PE), Fusion of eyes and accumulation of blood near the yolk area. Upon a close examination, the 1% ethanol-treated group showed an increase in the Yolk area, Pericardial Edema and accumulation of blood near the yolk area at 48 hpf in comparison to the control group as shown in Fig. 3. The fusion of eyes in the ethanol-treated group was observed at 72 hpf. When embryos were co-administered with 10 µM of benzydamine, the size of the yolk area was observed as a control along with a decrease in the pericardial edema as compared to ethanol treated group. However, 15 µM of benzydamine co-administration abolished the teratogenic effects of ethanol at 96 hpf which further confirms the protective effect of benzydamine against ethanol-induced condition.

### Oxidative stress induced by ethanol regulated after benzydamine co-exposure

**Fig. 4:**
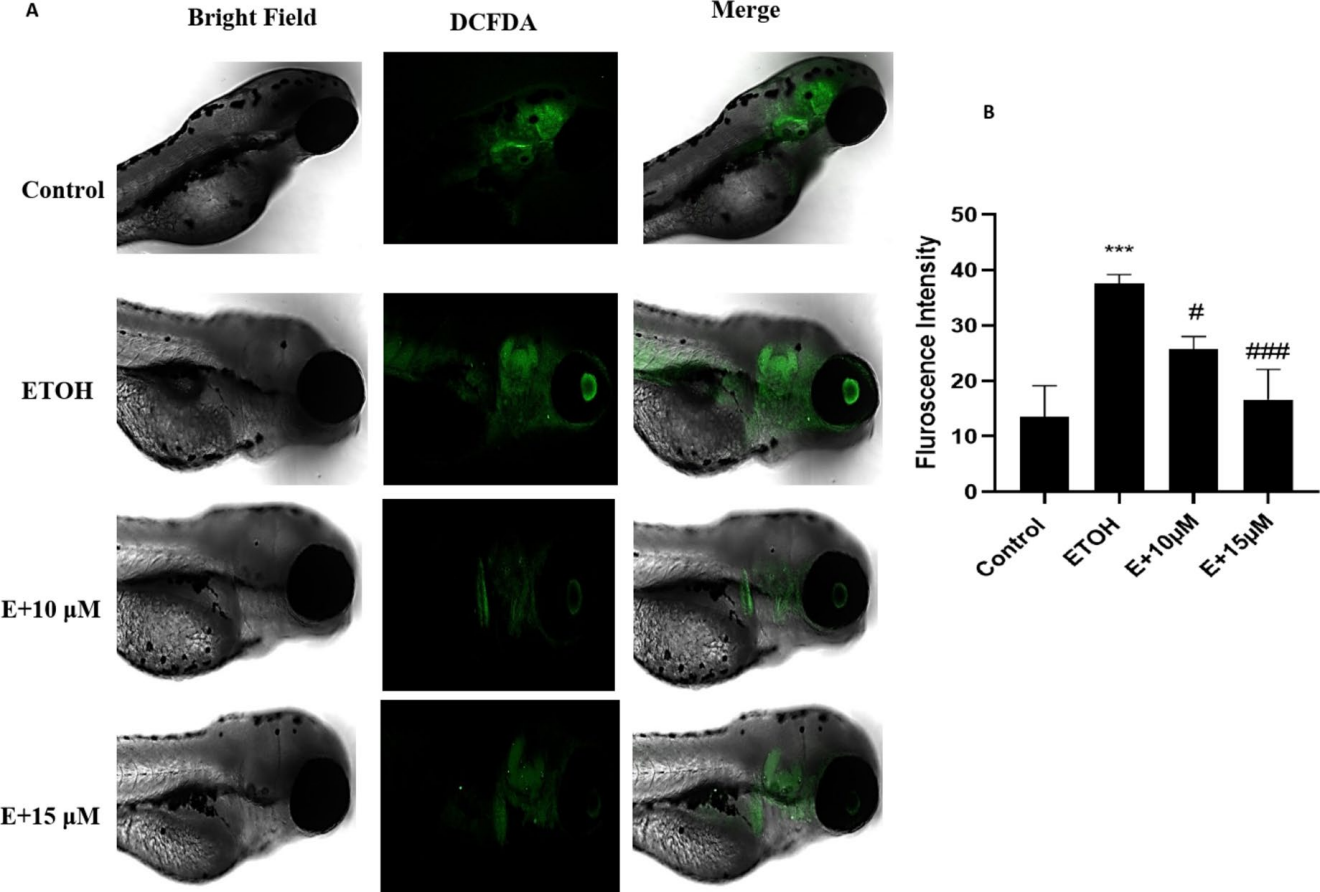
Reactive Oxygen Species (ROS) generation after exposure to 1% ETOH and in combination with benzydamine. (A) ROS generation (B) Fluorescence Intensity measured using image J. Data represented as mean ± SD of replicate samples. *** *P* < 0.001 vs. control group;. #*P* < 0.05 vs. ethanol-treated group, ###*P* < 0.001 vs. ethanol-treated group. Magnification 20X. Scale Bar- 200 μm.

The generation of Reactive oxygen species (ROS) during ethanol metabolism is one of the key indicators of oxidative stress. The microscopic images obtained from our study showed an increase in the fluorescence compared to control after 1% ethanol treatment which was reversed with the administration of benzydamine (10 and 15 µM) (Fig. 4A). The fluorescence intensity of the same was quantified using image J (Fig. 4B). 1% ethanol significantly increased ROS generation and the administration of 15 µM of benzydamine showed comparatively better reduction in the fluorescence than 10 µM of benzydamine.

**Fig. 5:**
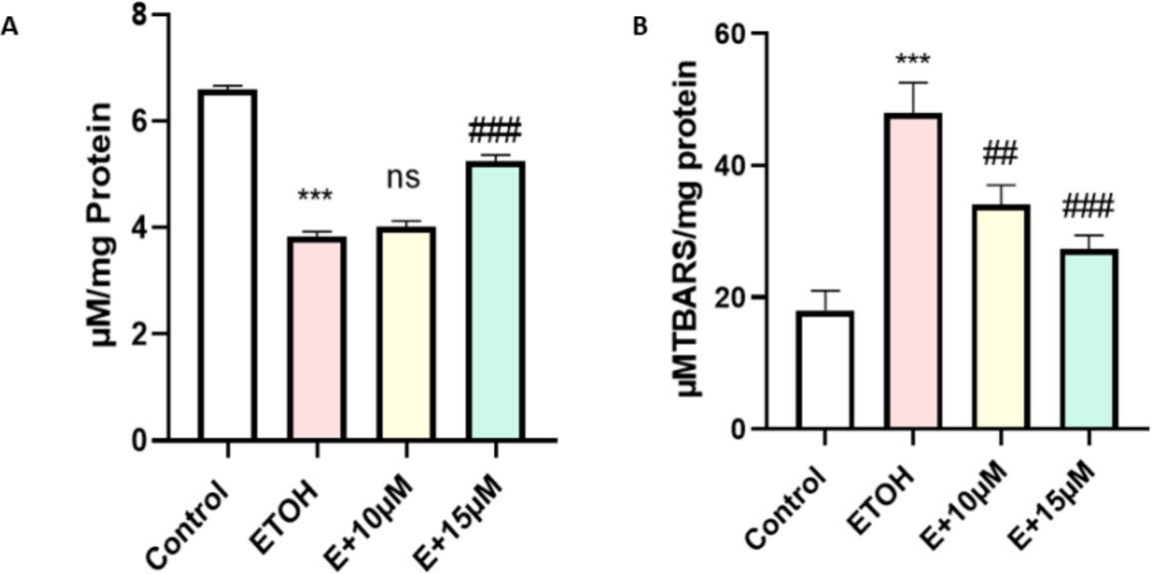
Effect of ETOH and in combination with benzydamine on cellular oxidative stress markers. (A) GSH (B) LPO. Data represented as mean ± SD of replicate samples. *** *P* < 0.001 vs. control group;. ##*P* < 0.01 vs. ethanol-treated group, ###*P* < 0.001 vs. ethanol-treated group.

Generation of ROS has several impacts on cells, increase in lipid peroxidation due to free radical attack towards lipid molecules is another subtle key marker during oxidative stress. A significant increase in the LPO was observed after 1% of ethanol treatment (*P* < 0.001). We found dose-dependent decrease in the MDA production with benzydamine co-treatment in zebrafish embryos (Fig. 5B).

Another classical biomarker involved during oxidative stress is reduced glutathione (GSH). We measured the GSH level in the zebrafish embryos as shown in Fig. 5A. Introduction of 1% ethanol to the embryos has significantly reduced cellaular ghutathione level. But interestingly we couldn’t find any significant change in GSH level after 10 µM of benzydamine treatment in comparison to ethanol. However, 15 µM benzydamine treatment re-established the GSH level in the ethanol-treated zebrafish group.

### Benzydamine stabilize ethanol metabolizing enzymes’ gene expression in zebrafish embryos at 48hpf

**Fig. 6:**
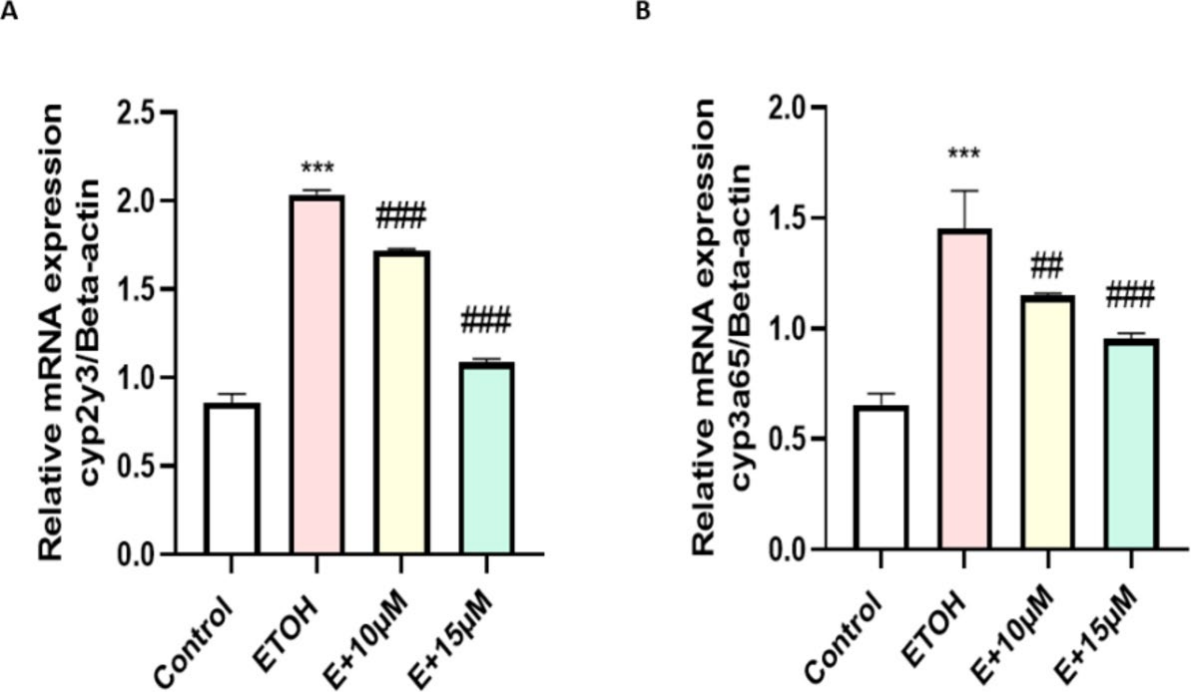
Effect of ETOH and in combination with benzydamine on ethanol oxidizing enzymes. (A) *cyp2y3* (B) *cyp3a65*. Data represented as mean ± SD of replicate samples. *** *P* < 0.001 vs. control group; ##*P* < 0.01 vs. ethanol-treated group, ###*P* < 0.001 vs. ethanol-treated group.

To assess the direct effect of benzydamine on ethanol metabolism and toxicity we evaluated the expression of two major metabolizing enzymes *cyp2y3* and *cyp3a65*. Whereas, co-treatment with benzydamine ( 10 and 15 µM) significantly decreased the expression of these two enzymes to a minimal level as depicted in Fig. 6.

### Histopathology study

**Fig. 7:**
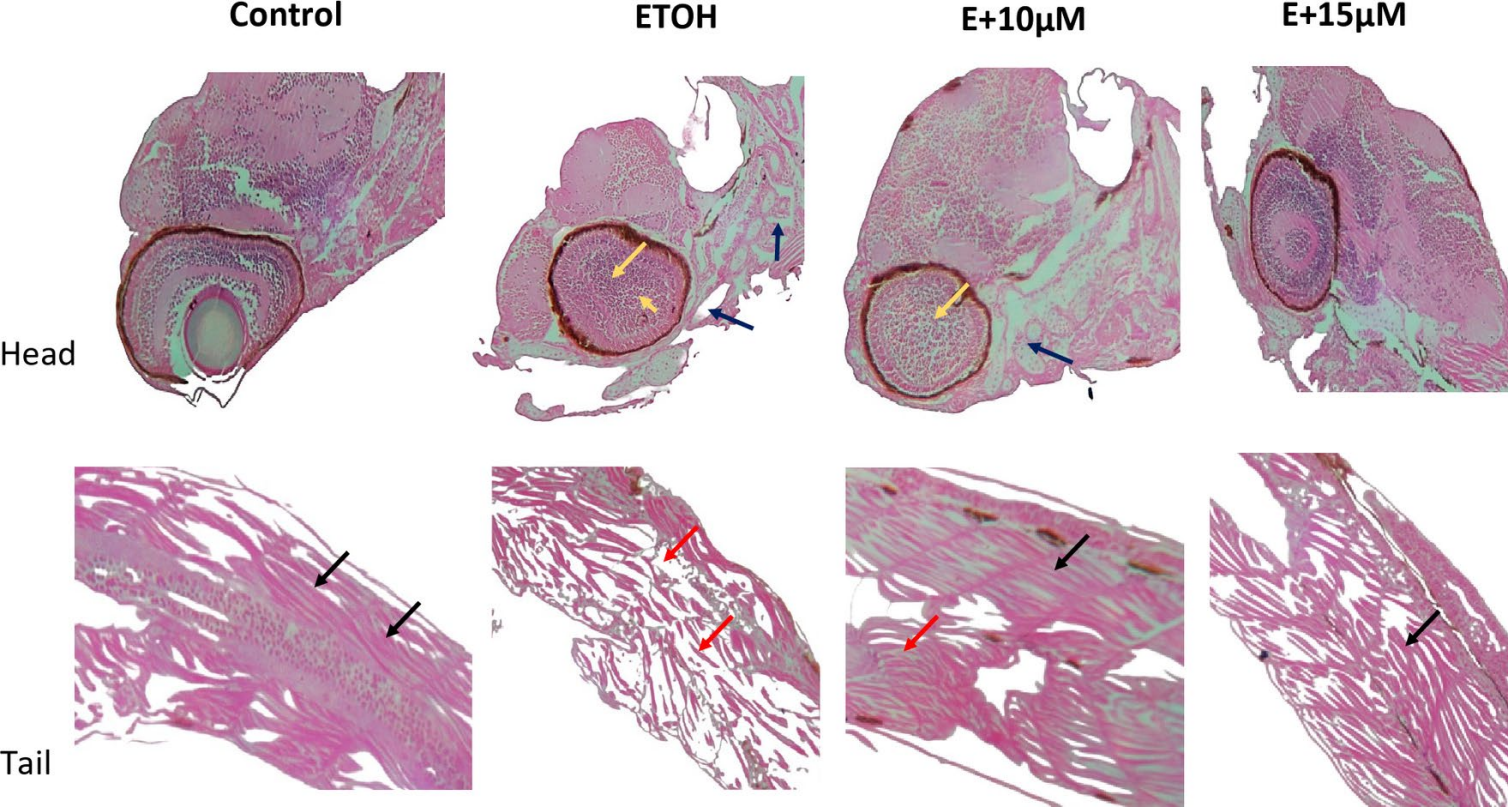
Histopathological images of 72 hpf zebrafish embryos treated with 1% ETOH and in combination with benzydamine. Image Magnification 10X. Black Arrow represents muscle fiber with normal morphology, Red represents the muscle fiber alterations. Blue arrow represents the apoptosis in the brain. Yellow arrow represents apoptosis in the eye area.

The teratogenic effect of ethanol was confirmed by histopathological examination of zebrafish embryos exposed to 1% ethanol. As shown in Fig. 7 control failed to show any notable variation in the histology whereas 1% ethanol appeared to have muscle fibre alteration (red arrow) besides visible apoptosis in the brain (blue arrow) and eye area (yellow arrow). Treatment with 10 µM benzydamine has reduced the amount of alteration in the embryos however 15 µM appeared to be more protective towards ethanol exposure.

### AO staining

**Fig. 8:**
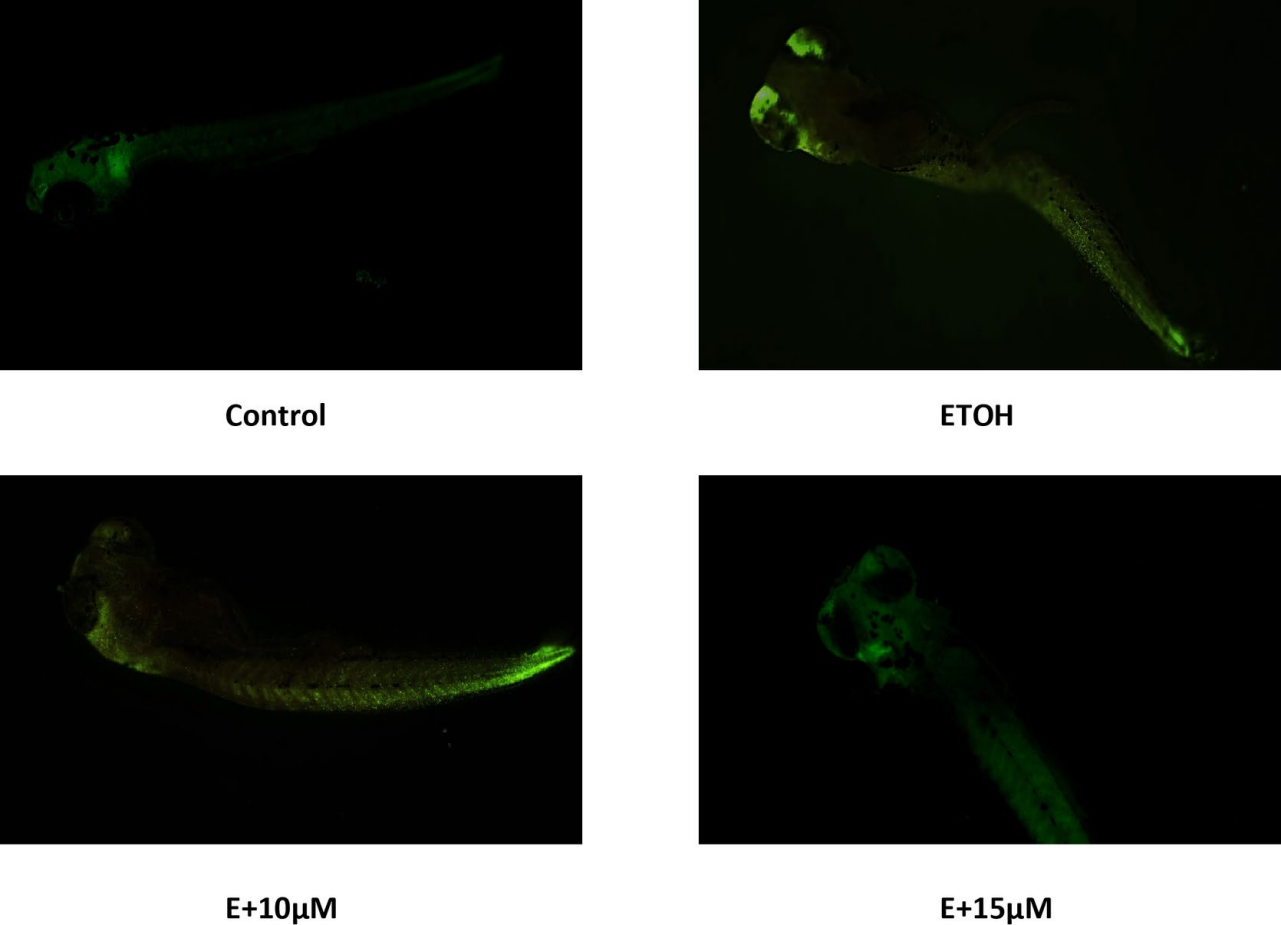
Ethanol-induced apoptosis analysed using AO staing in zebrafish embryos.

We have evaluated apoptsis induction due to ethanol exposure by AO staining. Ethnaol 1% significantly increased apoptisis in zebrafish embryos nearly brain and eye region followed by tail. However, treatment with benzydamine has decreased fluroscence intensity in zebrafish embryos thus suggesting its protective effect against ethanol-induced apoptsis as depicted in Fig. 8.

## Discussion

In humans, drinking alcohol during pregnancy can cause FASD in the developing embryos because of their ability to pass through the placental barrier^25,26^. Therefore, this study aims to assess benzydamine (renowned NASID class of drug) administration as a possible novel therapy against ethanol-induced teratogenesis in zebrafish embryos through the analysis of various biochemical and morphological parameters. Precisely, it has been demonstrated in our previous study that benzydamine positively stabilizes the redox system in RAW 264.7 murine macrophages when exposed to ethanol and thus can serve as a potential antioxidant and protects against ethanol-induced teratogenicity in the course of zebrafish embryo development^21^.

We here shown that 1% ethanol significantly increased the number of embryos with malformation including an increase in the yolk area, reduction in eye size and pericardial edema as depicted in Fig. 3, which are major hallmarks of FASD in zebrafish along with a significant decrease in the number of live embryos throughout the study, meanwhile, co-exposure of ethanol and benzydamine ( 10 and 15 µM) for 24 h protected the embryos from drastic developmental malformation as well as partially decreased the severity of FASD. Importantly, histopatholigical analysis revealed the disease severity between control and 1% ethanol-induced group including differentiation between muscle fiber alreatation mainly at the tail region and cellular damage due to increase number of apoptosis near brain and eye as shown in Figs. 7 and 8. The build-up of excessive amounts of intercellular watery fluids in these early life stages has been observed in different species and exposure regimes and linked to osmoregulatory abnormalities and/or changes in heart function^27,28^. However, the complicated and spatiotemporal genetic cascade that has been linked to the etiology of microphthalmia phenotypes is still poorly understood^28^. Cardiac precursors in zebrafish embryos start to discriminate at 16 hpf^29^. Thus, it is reasonable to surmise that osmoregulatory alterations may be connected to the swelling near heart and yolk sac area which was seen following a 1% ethanol exposure. Interestingly, teleost embryos’ development of functional osmoregulatory organs rely on ionocytes, or mitochondrion-rich cells, found in the yolk-sac membrane^30^, thus the development of these organs occurs only after 48hpf^31^. The possible meachnism contributes to this severe damage mainly due to excess amount of ROS generation during ethanol metabilosm. Benzydamine was shown to have antioxidant properties by acting as a ROS scavenger from infiltered neutrophils during the inflammation process^32^ thus co-exposure at a higher concentration (15 µM) decreased the generation of ROS and reversed the cellular damage.

Research demonstrated that embryos treated with ethanol (at a dosage of 1%), between 3 and 24 hpf—a stage akin to the first trimester of human gestation and the prenatal stages of mouse and rat development display substantial developmental abnormalities^9,33^. The deleterious effects of ethanol arise due to the generation of ROS such as hydrogen peroxide (H_2_O_2_) and superoxide ions (O_2_^−^)^34^. Several antioxidant systems, such as glutathione, work to maintain their levels within reasonable bounds to support various developmental processes during embryogenesis by regulating redox status^35^. The antioxidant mechanism has been altered through excess generation of ROS along with depletion in GSH during teratogenesis^36^. Our current investigation correlates with previous studies where 1% ethanol exposure significantly increased the generation of ROS in zebrafish embryos, along with decreased intercellular glutathione levels as reported previously. The generation of ROS and acetaldehyde (the first metabolite of ethanol metabolism) can activate inflammatory factor NF-κB which further increases inflammation inside the cells^4^. Also, an increase in ROS generation is linked to the muscle fibre alteration during zebrafish embryos development via the NF-κB pathway^37^. Our previous study has shown that benzydamine reduced NF-κB translocation during ethanol exposure^21^ thus suggesting its positive impact on changes in the muscle fiber alteration shown in histopathology data after co-exposed with ethanol.

However, The excess amount of ROS generation during ethanol metabolism happens majorly by a class of enzyme named cytochrome 450 CYP2 which converts ethanol to its primary metabolite acetaldehyde by producing a larger number of ROS inside mitochondria due to an overload of ethanol inside the cells. In zebrafish, the ortholog of cytochrome 450 CYP2, Cytochrome P450, family 2, subfamily Y, polypeptide 3 (*cyp2y3)*is required for ethanol metabolism^38^. Notably, our data indicated that benzydamine treatment brought down the expression of *cyp2y3* when compared with embryos treated with 1% ethanol for 24 h. Similarly, cytochrome P450, family 3, subfamily A, polypeptide 65 *(cyp3a65)* which shares an ortholog to cytochrome P450, family 3, subfamily A (*cyp3a*) plays a vital role during xenobiotics such as drugs, pesticides and substrate like ethanol, methanol metabolism^39^. Previously it was shown not only *cyp2y3* but also *cyp3a*plays an provital role during ethanol metabolism and removal of toxic by product like acetaldehyde and acetate^40^. In our study, the expression of *cyp3a65* has increased significantly after 1% ethanol induction which stabilized after benzydamine treatment as shown in Fig. 6B. Our current investigation for the frist time showed benzydamine regulates the expression of these two major ethanol and substarte metabolizing enzymes in the cellular level after ethanol exposure. Thus this current investigation put an feather on the cap of benzydamine and showed the reduction in ROS is not only via regulating neutrophils but also by regulating major ethanol metabolizing enzymes.

Consequently, an increase in the generation of ROS alters organelle or cellular membranes leading to lipid peroxidation (LPO) because of their high content of polyunsaturated fatty acids (PUFA) which may also function as a signal for programmed cell death. It is also possible that this could be due the increased production of pro-inflammatory mediators prostaglandin, and thromboxane thus promoting neurodegeneration^41^. Our histopathology data has shown an increase in cellular apoptosis near the brain and eye region which may be due to an excess amount of prostaglandin synthesis associated with neurodegeneration. It is also likely that benzydamine by blocking prostaglandin synthesis^42^ shows a protective effect against FSAD which may in turn be the reason for the decrease in LPO and cellular apoptosis. The above result suggests that part of the protective effect of benzydamine may be mediated by lowering of intracellular ROS production at the inflammatory site. Combining the above observations, we can state that treatment with benzydamine may be advantageous due to its positive impact on ethanol metabolism and by lowering the toxic substances by regulating the expression of *cyp2y3* and *cyp3a*.

In conclusion, benzydamine appeared to be a promising drug molecule against ethanol-induced teratogenicity in zebrafish embryos by ameliorating ethanol-induced developmental impairments phenotypically by stabilizing redox homeostasis. A mechanistic way of approach to this study sheds light on understanding their molecular interactions and bindings with the targeted proteins.

## Electronic supplementary material

Below is the link to the electronic supplementary material.

Supplementary Material 1

## Data Availability

All data generated or analysed during this study are included in this published article.

## References

[1] Haberstick, B. C. et al. Prevalence and correlates of alcohol and cannabis use disorders in the United States: results from the national longitudinal study of adolescent health., *Drug Alcohol Depend.*, vol. 136, pp. 158–161, Mar. (2014). 10.1016/j.drugalcdep.2013.11.022 10.1016/j.drugalcdep.2013.11.022 PMC3963405 24440049

[2] Derme, M. et al. Oxidative stress in a mother consuming alcohol during pregnancy and in her newborn: A case report. *Antioxidants*10.3390/antiox12061216 (2023). 37371946 10.3390/antiox12061216 PMC10295254

[3] Pinheiro-da-Silva, J. & Luchiari, A. C. Embryonic ethanol exposure on zebrafish early development., *Brain Behav.*, vol. 11, no. 6, p. e02062, Jun. (2021). 10.1002/brb3.2062 10.1002/brb3.2062 PMC8213935 33939334

[4] Bilotta, J., Barnett, J. A., Hancock, L. & Saszik, S. Ethanol exposure alters zebrafish development: a novel model of fetal alcohol syndrome. *Neurotoxicol Teratol*. **26** (6), 737–743. 10.1016/j.ntt.2004.06.011 (2004). 15451038 10.1016/j.ntt.2004.06.011

[5] Medicine, I. *Fetal Alcohol Syndrome: Diagnosis, Epidemiology, Prevention, and Treatment* (National Academies, 1996).

[6] Kaufman, M. H. The teratogenic effects of alcohol following exposure during pregnancy, and its influence on the chromosome constitution of the pre-ovulatory egg. *Alcohol Alcohol*. **32** (2), 113–128. 10.1093/oxfordjournals.alcalc.a008245 (1997). 9105505 10.1093/oxfordjournals.alcalc.a008245

[7] Fiore, M. et al. Markers of neuroinflammation in the serum of prepubertal children with fetal alcohol spectrum disorders. *CNS Neurol. Disord Drug Targets*. **21** (9), 854–868. 10.2174/1871527320666211201154839 (2022). 34852752 10.2174/1871527320666211201154839

[8] Abel, E. L. Behavioral teratology of alcohol., *Psychol. Bull.*, vol. 90, no. 3, pp. 564–581, Nov. (1981). 7197795

[9] Fernandes, P., Monteiro, S. M., Venâncio, C. & Félix, L. 24-Epibrassinolide protects against ethanol-induced behavioural teratogenesis in zebrafish embryo. *Chem. Biol. Interact.* **328**, 109193. 10.1016/j.cbi.2020.109193 (2020). 32668205 10.1016/j.cbi.2020.109193

[10] Sarmah, S. & Marrs, J. A. Complex cardiac defects after ethanol exposure during discrete cardiogenic events in zebrafish: prevention with folic acid., *Dev. Dyn. an Off. Publ. Am. Assoc. Anat.*, vol. 242, no. 10, pp. 1184–1201, Oct. (2013). 10.1002/dvdy.24015 10.1002/dvdy.24015 PMC4260933 23832875

[11] Jiang, Q. et al. Folic acid supplement rescues ethanol-induced developmental defects in the zebrafish embryos. *Acta Biochim. Biophys. Sin (Shanghai)*. **52** (5), 536–545. 10.1093/abbs/gmaa030 (May 2020). 32369106 10.1093/abbs/gmaa030

[12] Kalueff, A. V., Stewart, A. M. & Gerlai, R. Zebrafish as an emerging model for studying complex brain disorders. *Trends Pharmacol. Sci.* **35** (2), 63–75. 10.1016/j.tips.2013.12.002 (2014). 24412421 10.1016/j.tips.2013.12.002 PMC3913794

[13] Joya, X., Garcia-Algar, O., Vall, O. & Pujades, C. Transient exposure to ethanol during zebrafish embryogenesis results in defects in neuronal differentiation: an alternative model system to study FASD. *PLoS One*. **9** (11), e112851. 10.1371/journal.pone.0112851 (2014). 25383948 10.1371/journal.pone.0112851 PMC4226617

[14] Fu, J., Han, N., Jiao, J. & Shi, G. Effects of Embryonic Exposure to Ethanol on Zebrafish Survival, Growth Pattern, Locomotor Activity and Retinal Development., *Altern. Ther. Health Med.*, vol. 27, no. 5, pp. 120–128, Sep. (2021). 34582364

[15] Manikandan, P., Sarmah, S. & Marrs, J. A. Ethanol effects on early developmental stages studied using the zebrafish. *Biomedicines* **10** (10). 10.3390/biomedicines10102555 (Oct. 2022). 36289818 10.3390/biomedicines10102555 PMC9599251

[16] Pushpakom, S. et al. Drug repurposing: progress, challenges and recommendations. *Nat. Rev. Drug Discov*. **18** (1), 41–58. 10.1038/nrd.2018.168 (2019). 30310233 10.1038/nrd.2018.168

[17] Passali, D. et al. Benzydamine hydrochloride for the treatment of sore throat and irritative/inflammatory conditions of the oropharynx: a cross-national survey among pharmacists and general practitioners. *BMC Prim. Care*. **23** (1), 154. 10.1186/s12875-022-01762-3 (2022). 35715725 10.1186/s12875-022-01762-3 PMC9205545

[18] Ősz, B. E., Jîtcă, G., Sălcudean, A., Rusz, C. M. & Vari, C. E. Benzydamine—An affordable Over-the-Counter drug with psychoactive properties—From chemical structure to possible Pharmacological properties. *Pharmaceuticals*. 10.3390/ph16040566 (2023). 37111323 10.3390/ph16040566 PMC10144213

[19] Molina-Holgado, E., Ortiz, S., Molina‐Holgado, F. & Guaza, C. Induction of COX‐2 and PGE2 biosynthesis by IL‐1β is mediated by PKC and mitogen‐activated protein kinases in murine astrocytes. *Br. J. Pharmacol.* **131** (1), 152–159 (2000). 10960082 10.1038/sj.bjp.0703557 PMC1572306

[20] Cho, W. & Choe, J. Prostaglandin E2 stimulates COX-2 expression via mitogen-activated protein kinase p38 but not ERK in human follicular dendritic cell-like cells. *BMC Immunol.* **21**, 1–8 (2020). 32303181 10.1186/s12865-020-00347-y PMC7165408

[21] Dasgupta, T. & Manickam, V. Benzydamine hydrochloride ameliorates ethanol-induced inflammation in RAW 264.7 macrophages by stabilizing redox homeostasis, *Asian Pac. J. Trop. Biomed.*, vol. 14, no. 2, [Online]. (2024). Available: https://journals.lww.com/aptb/fulltext/2024/14020/benzydamine_hydrochloride_ameliorates.4.aspx

[22] Wojtala, A. et al. Methods to monitor ROS production by fluorescence microscopy and fluorometry. *Methods Enzymol.* **542**, 243–262. 10.1016/B978-0-12-416618-9.00013-3 (2014). 24862270 10.1016/B978-0-12-416618-9.00013-3

[23] Umamaheswari, S., Priyadarshinee, S., Bhattacharjee, M., Kadirvelu, K. & Ramesh, M. Exposure to polystyrene microplastics induced gene modulated biological responses in zebrafish (Danio rerio). *Chemosphere* **281**, 128592. 10.1016/j.chemosphere.2020.128592 (2021). 33077188 10.1016/j.chemosphere.2020.128592

[24] Rahman, I., Kode, A. & Biswas, S. K. Assay for quantitative determination of glutathione and glutathione disulfide levels using enzymatic recycling method. *Nat. Protoc.* **1** (6), 3159–3165. 10.1038/nprot.2006.378 (2006). 17406579 10.1038/nprot.2006.378

[25] Gilbert-Barness, E. Teratogenic causes of malformations. *Ann. Clin. Lab. Sci.* **40** (2), 99–114 (2010). 20421621

[26] Lipinski, R. J. et al. Ethanol-induced face-brain dysmorphology patterns are correlative and exposure-stage dependent. *PLoS One*. **7**, e43067. 10.1371/journal.pone.0043067 (2012). no. 8. 22937012 10.1371/journal.pone.0043067 PMC3425589

[27] Incardona, J. P. & Scholz, N. L. The influence of heart developmental anatomy on cardiotoxicity-based adverse outcome pathways in fish. *Aquat. Toxicol.* **177**, 515–525. 10.1016/j.aquatox.2016.06.016 (2016). 27447099 10.1016/j.aquatox.2016.06.016

[28] Richardson, R., Tracey-White, D., Webster, A. & Moosajee, M. The zebrafish eye—a paradigm for investigating human ocular genetics. *Eye* **31** (1), 68–86. 10.1038/eye.2016.198 (2017). 27612182 10.1038/eye.2016.198 PMC5233929

[29] Glickman, N. S. & Yelon, D. Cardiac development in zebrafish: coordination of form and function. *Semin Cell. Dev. Biol.* **13** (6), 507–513. 10.1016/S1084952102001040 (2002). 12468254 10.1016/s1084952102001040

[30] Varsamos, S., Nebel, C. & Charmantier, G. Ontogeny of osmoregulation in postembryonic fish: A review. *Comp. Biochem. Physiol. Part. Mol. Integr. Physiol.* **141** (4), 401–429. 10.1016/j.cbpb.2005.01.013 (2005). 10.1016/j.cbpb.2005.01.013 16140237

[31] Esaki, M. et al. Mechanism of development of ionocytes rich in vacuolar-type H+-ATPase in the skin of zebrafish larvae. *Dev. Biol.* **329** (1), 116–129. 10.1016/j.ydbio.2009.02.026 (2009). 19268451 10.1016/j.ydbio.2009.02.026 PMC2751791

[32] Nicolatou-Galitis, O., Bossi, P., Orlandi, E. & Bensadoun, R. J. The role of benzydamine in prevention and treatment of chemoradiotherapy-induced mucositis. *Support Care Cancer*. **29**, 5701–5709 (2021). 33649918 10.1007/s00520-021-06048-5 PMC8410701

[33] Bilotta, J., Saszik, S., Givin, C. M., Hardesty, H. R. & Sutherland, S. E. Effects of embryonic exposure to ethanol on zebrafish visual function. *Neurotoxicol Teratol*. **24** (6), 759–766. 10.1016/S0892-0362(02)00319-7 (2002). 12460658 10.1016/s0892-0362(02)00319-7

[34] Linhart, K., Bartsch, H. & Seitz, H. K. The role of reactive oxygen species (ROS) and cytochrome P-450 2E1 in the generation of carcinogenic etheno-DNA adducts. *Redox Biol.* **3**, 56–62. 10.1016/j.redox.2014.08.009 (2014). 25462066 10.1016/j.redox.2014.08.009 PMC4297928

[35] Masella, R., Di Benedetto, R., Varì, R., Filesi, C. & Giovannini, C. Novel mechanisms of natural antioxidant compounds in biological systems: involvement of glutathione and glutathione-related enzymes. *J. Nutr. Biochem.* **16** (10), 577–586. 10.1016/j.jnutbio.2005.05.013 (2005). 16111877 10.1016/j.jnutbio.2005.05.013

[36] Hansen, J. M., Jacob, B. R. & Piorczynski, T. B. Oxidative stress during development: Chemical-induced teratogenesis. *Curr. Opin. Toxicol.* **7**, 110–115. 10.1016/j.cotox.2017.11.003 (2018).

[37] Wen, W. et al. Alcohol induces zebrafish skeletal muscle atrophy through HMGB1/TLR4/NF-κB signaling. *Life (Basel Switzerland)*. **12** (8). 10.3390/life12081211 (2022). 36013390 10.3390/life12081211 PMC9410481

[38] Lin, H. et al. Naringenin inhibits alcoholic injury by improving lipid metabolism and reducing apoptosis in zebrafish larvae. *Oncol. Rep.* **38** (5), 2877–2884 (2017). 29048675 10.3892/or.2017.5965

[39] Tseng, H. P., Hseu, T. H., Buhler, D. R., Wang, W. D. & Hu, C. H. Constitutive and xenobiotics-induced expression of a novel CYP3A gene from zebrafish larva. *Toxicol. Appl. Pharmacol.* **205** (3), 247–258 (2005). 15922010 10.1016/j.taap.2004.10.019

[40] Niemelä, O. et al. Induction of cytochrome P450 enzymes and generation of protein-aldehyde adducts are associated with sex‐dependent sensitivity to alcohol‐induced liver disease in micropigs. *Hepatology* **30** (4), 1011–1017 (1999). 10498654 10.1002/hep.510300413

[41] Que, X. et al. Oxidized phospholipids are Proinflammatory and proatherogenic in hypercholesterolaemic mice. *Nature* **558** (7709), 301–306 (2018). 29875409 10.1038/s41586-018-0198-8 PMC6033669

[42] Modéer, T. & Yucel-Lindberg, T. Benzydamine reduces prostaglandin production in human gingival fibroblasts challenged with interleukin-1 beta or tumor necrosis factor alpha., *Acta Odontol. Scand.*, vol. 57, no. 1, pp. 40–45, Feb. (1999). 10.1080/000163599429093 10.1080/000163599429093 10207535

