# Supplementary material for: Benzydamine rescues ethanol-induced teratogenesis in zebrafish FASD model
Source: Sci Rep. 2025 Mar 17;15:9066. doi: 10.1038/s41598-025-93539-8 (PMC11914598; doi:10.1038/s41598-025-93539-8)

## Slide 1
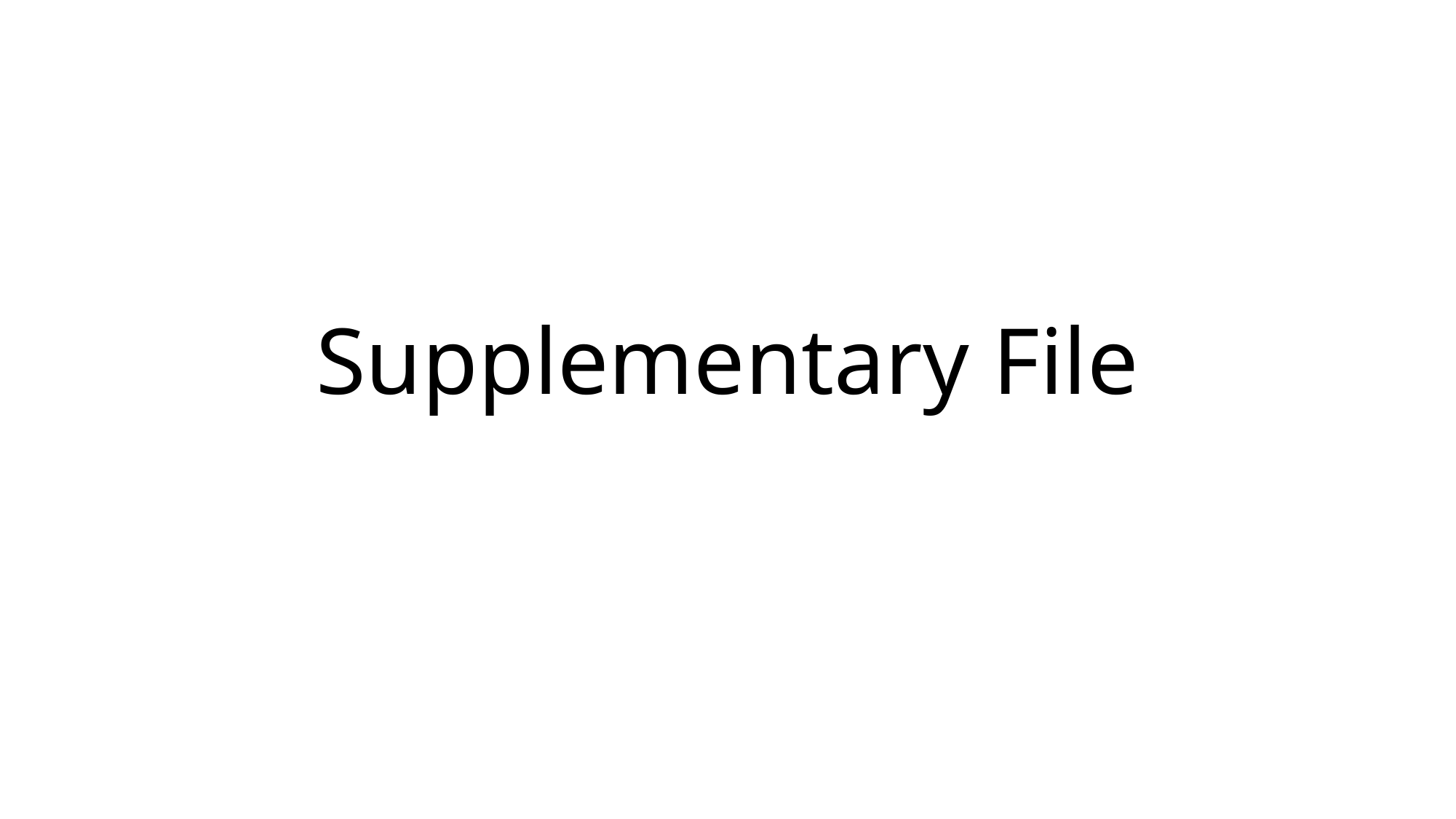

# Supplementary File

## Slide 2
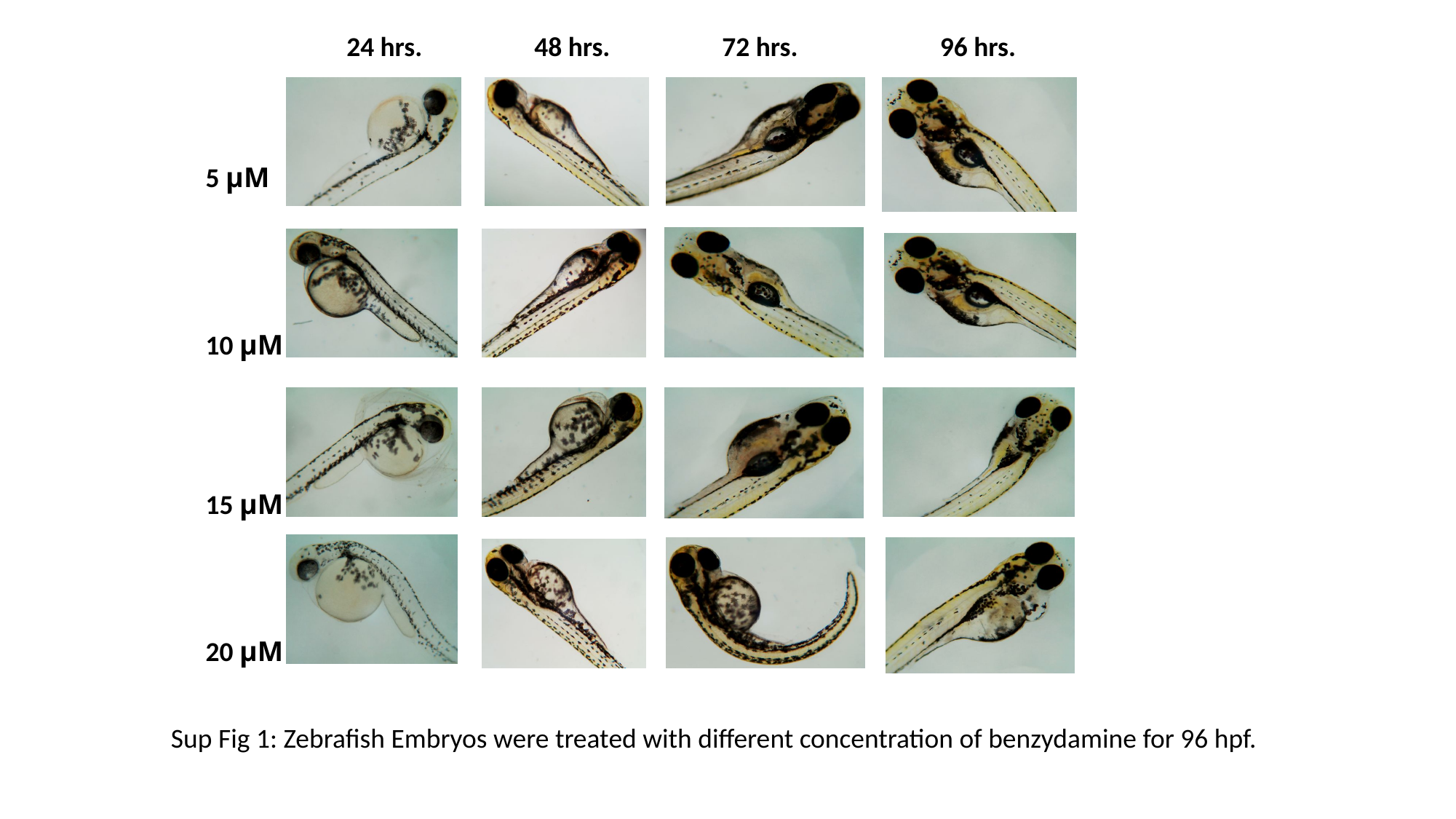

24 hrs.
48 hrs.
72 hrs.
96 hrs.
5 μM
10 μM
15 μM
20 μM
Sup Fig 1: Zebrafish Embryos were treated with different concentration of benzydamine for 96 hpf.

## Slide 3
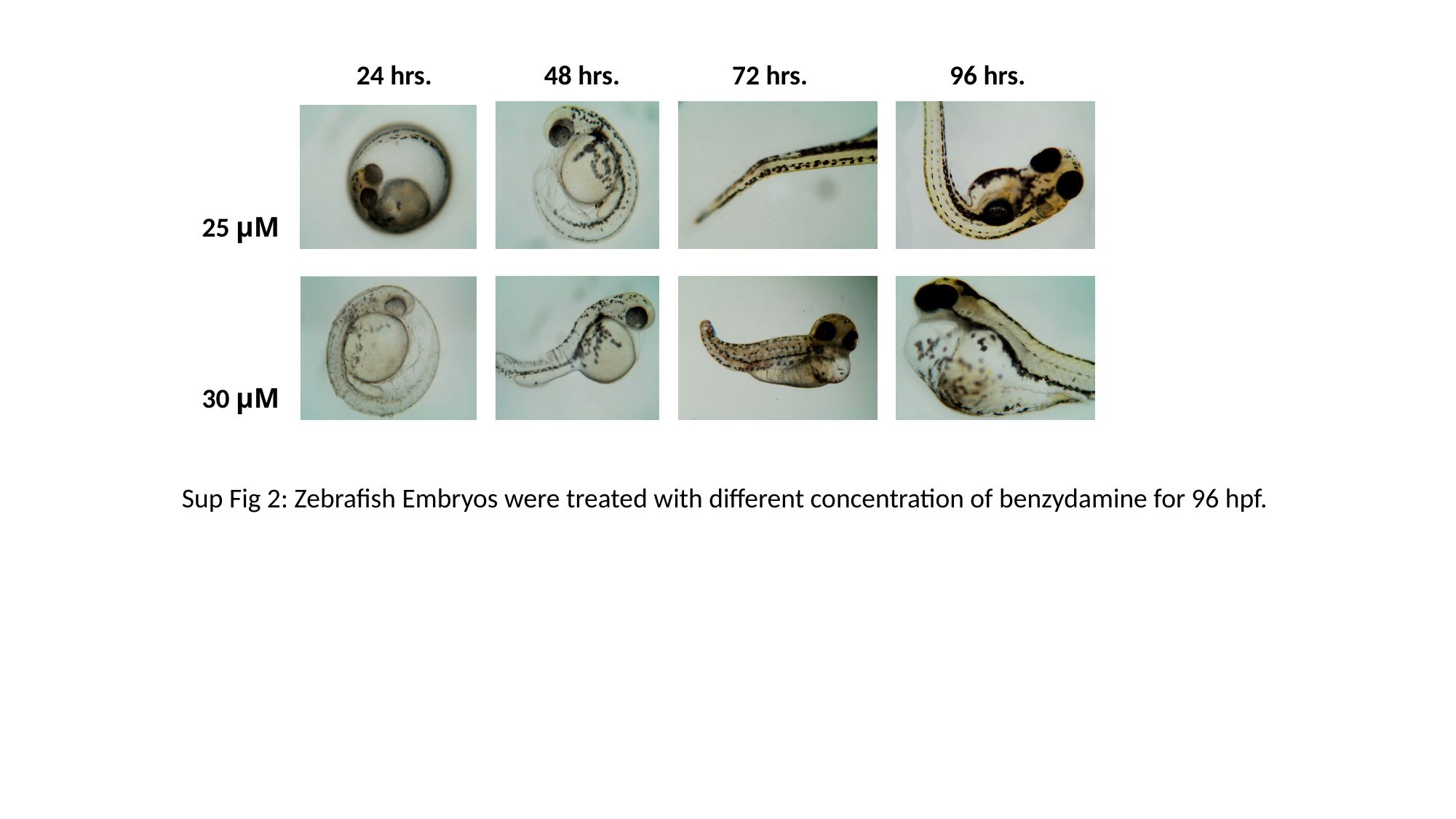

24 hrs.
48 hrs.
72 hrs.
96 hrs.
25 μM
30 μM
Sup Fig 2: Zebrafish Embryos were treated with different concentration of benzydamine for 96 hpf.

## Slide 4
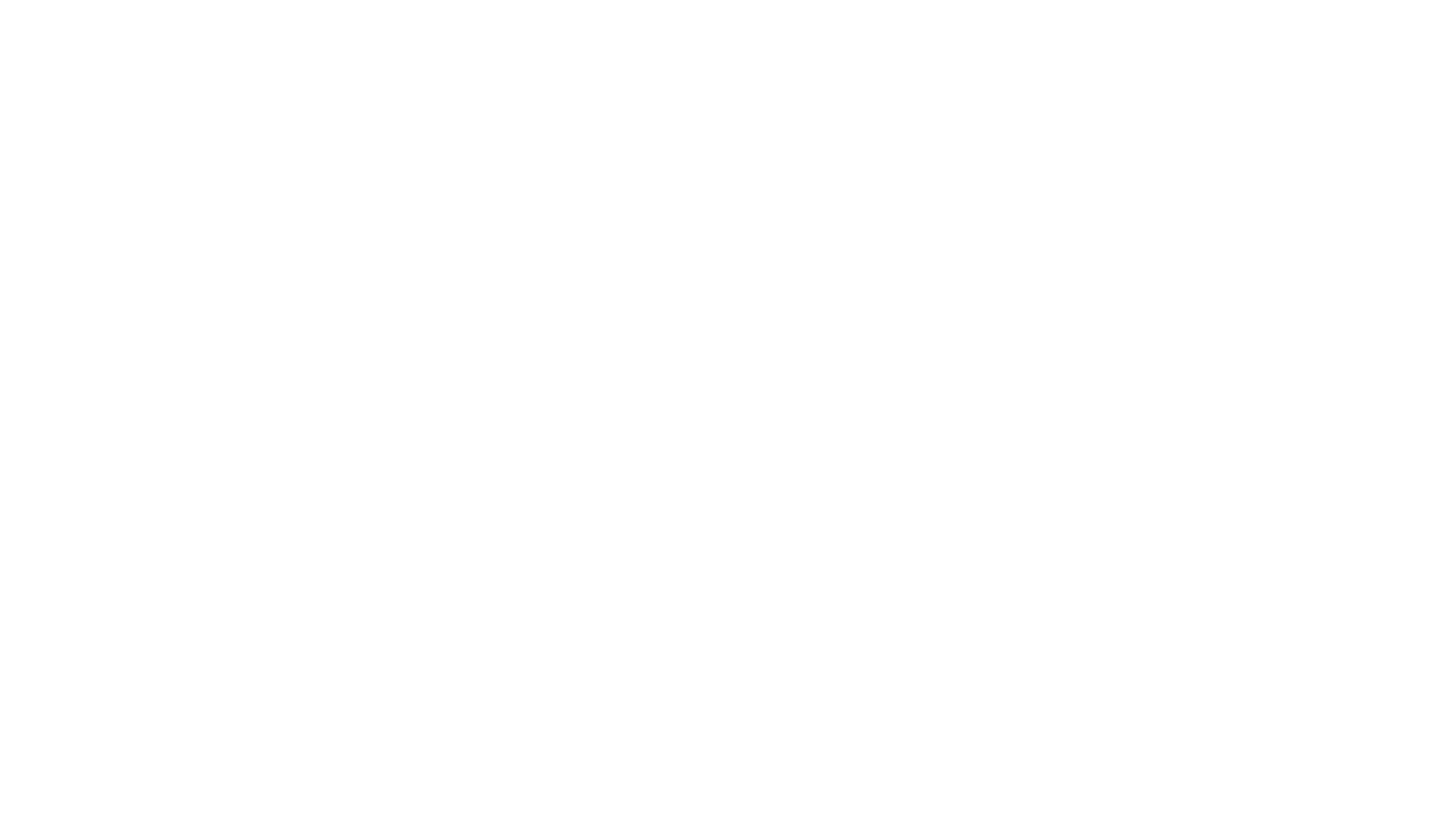

Supplement: Supplementary file 1 — Supplementary Material 1 [file 41598_2025_93539_MOESM1_ESM.pptx]
